# Anti NMDAR encephalitis masked by symptoms of postpartum depression

**DOI:** 10.1192/j.eurpsy.2023.2115

**Published:** 2023-07-19

**Authors:** S. Milanovic Hromin, A. Jelcic, R. Perincic, M. Komso, B. Barun, M. Grah, S. Zecevic Penic, S. Biloglav, I. Perincic, M. Juresko, B. Skific

**Affiliations:** 1 GENERAL HOSPITAL ZADAR, Zadar; 2 Ginekologija Dana d.o.o. for gynecology, obstetrics and consulting, Zagreb; 3 Private psychiatric office dr Robert Perincic, Zadar; 4 Department for neuroimunology of CNS, Clinical hospital center Zagreb; 5 Clinic for psychiatry Sveti Ivan, Zagreb, Croatia; 6 Neurological outpatient clinic, Clinic for psychiatry Sveti Ivan, Zagreb; 7 Department for psychiatry, GENERAL HOSPITAL ZADAR, Zadar, Croatia

## Abstract

**Introduction:**

Anti NMDAR encephalitis is a relatively common autoimmune encephalitis characterized by complex neuropsychiatric features and the presence of Immunoglobulin G antibodies against the NR1 subunit of the NMDA receptors in the central nervous system. It causes psychiatric features, confusion, memory loss and seizures followed by a movement disorder, loss of consciousness and changes in blood pressure, heart rate and temperature. Postpartum depression symptoms usually develop within the first few weeks after giving birth , but may begin earlier/during pregnancy / or later /up to a year after birth. They include: inability to sleep or sleeping too much, depressed mood or severe mood swing, difficulty bonding with your baby, withdrawing from family and friends, fatigue or loss of energy, feelings of shame, guilt or inadequacy, diminished ability to think clearly, concentrate or make decisions, anxiety and panic attacks, thoughts of harming yourself or your baby. Untreated may last for many months or longer.

**Objectives:**

Recent studies have highlighted the possibility that a subset of patients with first-onset severe psychiatric episodes might suffer from undiagnosed autoimmune encephalitis. The acute onset of severe atypical psychiatric symptoms in young female patients should raise the index of suspicion for anti-NMDA receptor encephalitis, particularly in the setting of neurological symptoms, including side effects of antipsychotic treatment.

**Methods:**

/

**Results:**

/

**Conclusions:**

Creating a therapeutic environment is an interdisciplinary clinical and theoretical approach to psychiatric treatment in hospital settings, the basic idea of which is that the entire environment has therapeutic potential. Psychodinamic knowledge and understanding of the process as well as principles of body-oriented psychotherapy may be of great importance in the treatment of these patients in addition to the use of pharmacotherapy.

**Disclosure of Interest:**

None Declared

